# Conjugated Linoleic Acid Treatment Attenuates Cancerous features in Hepatocellular Carcinoma Cells

**DOI:** 10.1155/2022/1850305

**Published:** 2022-09-12

**Authors:** Zohre Miri-Lavasani, Shukoofeh Torabi, Roya Solhi, Bahareh Shokouhian, Parvaneh Afsharian, Zahra Heydari, Abbas Piryaei, Zahra Farzaneh, Nikoo Hossein-khannazer, Hamidreza Aboulkheyr Es, Ensieh Zahmatkesh, Andreas Nussler, Moustapha Hassan, Mustapha Najimi, Massoud Vosough

**Affiliations:** ^1^Department of Regenerative Medicine, Cell Science Research Center, Royan Institute for Stem Cell Biology and Technology, ACECR, Tehran, Iran; ^2^Department of Genetics, Reproductive Biomedicine Research Center, Royan Institute for Reproductive Biomedicine, ACECR, Tehran, Iran; ^3^Department of Applied Cell Sciences, Faculty of Basic Sciences and Advanced Medical Technologies, Royan Institute, Academic Center for Education, Culture and Research, Tehran, Iran; ^4^Department of Biology and Anatomical Sciences, School of Medicine, Shahid Beheshti University of Medical Sciences, Tehran, Iran; ^5^Department of Tissue Engineering and Applied Cell Sciences, School of Advanced Technologies in Medicine, Shahid Beheshti University of Medical Sciences, Tehran, Iran; ^6^Gastroenterology and Liver Diseases Research Center, Research Institute for Gastroenterology and Liver Diseases, Shahid Beheshti University of Medical Sciences, Tehran, Iran; ^7^School of Biomedical Engineering, University of Technology Sydney, 2007 Sydney, Australia; ^8^Siegfried Weller Institute for Trauma Research, University of Tübingen, 72076 Tübingen, Germany; ^9^Experimental Cancer Medicine, Institution for Laboratory Medicine, Karolinska Institute, Stockholm, Sweden; ^10^Laboratory of Pediatric Hepatology and Cell Therapy, Institute of Experimental and Clinical Research, Université Catholique de Louvain, Brussels, Belgium

## Abstract

**Background:**

A growing number of hepatocellular carcinoma (HCC), and recurrence frequency recently have drawn researchers' attention to alternative approaches. The concept of differentiation therapies (DT) relies on inducing differentiation in HCC cells in order to inhibit recurrence and metastasis. Hepatocyte nuclear factor 4 alpha (HNF4*α*) is the key hepatogenesis transcription factor and its upregulation may decrease the invasiveness of cancerous cells by suppressing epithelial-mesenchymal transition (EMT). This study aimed to evaluate the effect of conjugated linoleic acid (CLA) treatment, natural ligand of HNF4*α*, on the proliferation, migration, and invasion capacities of HCC cells in vitro. *Materials and Method.* Sk-Hep-1 and Hep-3B cells were treated with different doses of CLA or BIM5078 [1-(2′-chloro-5′-nitrobenzenesulfonyl)−2-methylbenzimidazole], an HNF4*α* antagonist. The expression levels of *HNF4a* and EMT related genes were evaluated and associated to hepatocytic functionalities, migration, and colony formation capacities, as well as to viability and proliferation rate of HCC cells.

**Results:**

In both HCC lines, CLA treatment induced *HNF4α* expression in parallel to significantly decreased EMT marker levels, migration, colony formation capacity, and proliferation rate, whereas BIM5078 treatment resulted in the opposite effects. Moreover, CLA supplementation also upregulated ALB, ZO1, and HNF4*α* proteins as well as glycogen storage capacity in the treated HCC cells.

**Conclusion:**

CLA treatment can induce a remarkable hepatocytic differentiation in HCC cells and attenuates cancerous features. This could be as a result of *HNF4a* induction and EMT inhibition.

## 1. Introduction

Hepatocellular carcinoma (HCC) is the most common primary liver cancer [1]. The global incidence of HCC is rising, and it is predicted that by 2030, this cancer will be one of the leading causes of cancer death worldwide. Patients with advanced tumors are offered different treatments, including systemic prescription-based therapies like sorafenib, regorafenib, and nivolumab to loco-regional ablation or resection [2–4]. Liver transplantation, immunotherapeutic and radionuclide-based approaches, and targeted molecular and gene therapy interventions are other advanced modalities [5, 6]. However, the high rate of tumor recurrence after treatment has led to a growing interest in developing innovative therapeutic approaches [7].

Liver chronic inflammation results in morphological changes and dedifferentiation of mature hepatocytes [8]. Poorly differentiated carcinoma cells have a worse prognosis and are more aggressive than the well-differentiated cells [9–12]. During epithelial-mesenchymal transition (EMT), epithelial parenchymal cells lose their cell-cell junctions and dissociate from each other and from the surrounding extracellular matrix (ECM) that results in the initiation of their migration and invasion [13, 14]. EMT is also associated with enhanced stem cell properties and drug resistance in cancer cells [15–17]. Recent findings indicated that EMT could be switched to mesenchymal-to-epithelial transition (MET) after modulating the gene expression pattern of EMT-related transcription factors (TFs) such as SNAIL, SLUG, TWIST1, and ZEB1 [18–20]. MET is one of the most essential mechanisms in regulating hepatocyte differentiation from definitive endoderm (DE); such process is orchestrated by hepatocyte nuclear factor 4 alpha (HNF4*α*) [21]. Understanding the cellular and molecular mechanisms of the hepatocytes dedifferentiation could provide necessary insights into differentiation therapy (DT) as a novel strategy in HCC treatment [22]. DT investigates the feasibility of converting the phenotype of cancerous cells toward a less aggressive and more differentiated one [23, 24]. Various strategies can induce differentiation of cancer cells through alteration of EMT molecular pathways including epigenetic alterations, miRNA-based methods to change the expression pattern, and TF-based mediated directed induction of signaling pathways [20]. Given that cellular differentiation is a continuous process regulated by different TFs, their application can be a practical approach to induce differentiation of cancer cells [25]. HNF4*α* is the key hepatogenesis TF which drives differentiation of stem and progenitor cells to mature hepatocytes and controls the acquisition of an epithelial phenotype [26–29]. In adult hepatocytes, *HNF4α* high expression is sustained in order to maintain the hepatocyte functions. HNF4*α* plays a pivotal role in the maintenance of epithelial/hepatocyte phenotype and regulates dynamic events of EMT by suppressing *snail*, the master regulator of EMT, and increasing E-cadherin in cancer cells [30, 31]. Downregulation of *HNF4α* has been demonstrated in HCC and its upregulation might accordingly suppress EMT and inhibit the progression of HCC [32, 33]. Several studies have shown that the induction of *HNF4α* activates the expression of various hepatocytic genes which enhances the differentiation of hepatocytes [34, 35].

The use of natural compounds as medications has gained significant attention [36, 37]. In 2009, a study showed that conjugated linoleic acid (CLA) acts as a natural intracellular ligand of HNF4*α* [38]. CLA is an 18-carbon essential free fatty acid with two double bonds, which were separated by a single bond that is why it is called conjugated. Natural forms of CLA are often found in ruminant products such as milk or cheese. The cis-9,trans-11 (c9,t11) is the common CLA isomer [39] and has shown its potential in treating some malignancies. Medical evidences have proved that the c9,t11 CLA isomer exerts its anticancer function by acting on apoptotic genes [40]. Despite many studies on the significance of *HNF4α* as an important target in preventing and treating liver malignancies, further investigations are still required to understand the mechanism and the correlation between CLA and *HNF4α* in regulating and inhibiting EMT. This study aimed to induce *HNF4a* expression by using c9,t11 isomer of CLA in order to reduce the cancerous phenotype (invasion and migration capacity) and proliferation rate of HCC cells.

## 2. Materials and Methods

### 2.1. Preparation of Chemical Compounds

The total amount of 50 mg conjugated linoleic acid (CLA, Sigma-Aldrich Co. Missouri, USA) was dissolved in 1780 *μ*l absolute ethanol to prepare 0.1 M CLA stock. To prepare 3.9 *μ*M BIM5078 and HNF4*α* antagonist (Sigma-Aldrich Co. Missouri, USA), 25 mg BIM5078 was dissolved in 1529 *μ*l DMSO. All stocks were stored at − 20 °C in dark.

### 2.2. Treatment of Hep-3B and Sk-Hep-1 cells with CLA

Two HCC cell lines taken from the Royan Institute cell bank were used in this study: Sk-Hep-1, an invasive endothelial hepatic carcinoma cell line, and Hep-3B, poorly differentiated primary liver cancer cells. No mutation in the *HNF4α* gene has been reported in both cell lines according to Broad Institute and CCLE databases.

Both cell lines were cultivated in Dulbecco's modified Eagle's medium (DMEM, high glucose, Life Technologies) supplemented with 10% fetal bovine serum (Life Technologies), 1% GlutaMAX™ (Life Technologies), 1% non-essential amino acid (Life Technologies), 1% penicillin-streptomycin (Life Technologies), and 0.1% 2-Mercapto ethanol (Sigma-Aldrich), at 37 °C and 5% CO2. The medium was changed every day, and passaging the cells was performed when 90% confluency was reached. The effects of treatments were evaluated twenty-four hours after cells plating, by using different concentrations of CLA or BIM in FBS free media for 48 h. The media were renewed every 24 h. After 48 h the samples were collected for analyses.

### 2.3. Cell Proliferation Assay

Sk-Hep-1 and Hep-3B cells were plated in 96-well plate (3 × 10^3^ cells/well) in 100 *μ*l culture medium and incubated overnight. The dose escalation data for both compounds at different time points were presented in Supplementary Figure 1(b). Accordingly, The cells were treated with different concentrations of CLA (Sk-Hep-1: 30 and 60 *μ*M, Hep-3B: 80 and 100 *μ*M) or BIM (Sk-Hep-1: 390 and 780 nM, Hep-3B: 780 and 1170 nM). To assess the cell proliferation rate in those different experimental conditions, Orangu™ kit (Cell Guidance Systems, Cambridge, UK) was used at 24 and 48 h after treatment, while metabolic activity was quantified by measuring light absorbance at 450 nm [41] and compared to the control nontreated group.

### 2.4. Colony Formation Assay

To evaluate the impact of CLA treatment on colony formation ability of Sk-Hep-1 and Hep-3B cells, about 10 cells/cm^2^ were seeded in a 6-well plate and cultured for four days in DMEM supplemented with 10% FBS. Then, Sk-Hep-1 and Hep-3B cells were, respectively, treated with 60 and 100 *μ*M CLA or BIM and 780 nM and 1170 nM in serum free medium. After 14 days, the emerged colonies were fixed by using 4% formaldehyde and stained by 0.5% crystal violet. Imaging was performed by inverted microscope (Olympus CKX41). The total number and the surface area of each colony were measured by ImageJ software (version 1.46).

### 2.5. RNA Extraction and Quantitative Real-Time PCR

Total RNA was extracted using RNA extraction kit (Macherey Nagel, KG, Duren, Germany), and the RNA quality was checked by gel electrophoresis (1.5 × 10^6^ cells per test). Synthesis of cDNA was performed by reverse transcription of 1 *μ*g total RNA using the cDNA synthesis kit (Life Technologies), according to the manufacturer's instructions. Quantitative real-time PCR assays were carried out using the StepOnePlus™ Real-Time PCR System (Applied Biosystems). Data analysis was performed with the Applied Biosystems StepOne software v2.3. The *C*_t_ values of target genes were normalized to *GAPDH* as reference gene and expressed as fold changes compared to the control group using the 2^−∆∆*CT*^ formula. The primer sequences used are listed in the Supplementary Table S1.

### 2.6. Migration Assay

The scratch assay was used to determine the impact of CLA treatment on the migration ability of Sk-Hep-1 and Hep-3B cells. A total number of 8 × 10^4^ cells/cm^2^ were seeded in a 6-well plate at 37 °C overnight. Cells were treated with Mitomycin C (5 *μ*g/ml, Sigma-Aldrich Co. Missouri, USA) to inhibit cell proliferation, 2 h before scratching the culture dishes with a cell scraper or pipette tip. The medium was refreshed, and cells were treated with CLA (Sk-Hep-1: 60 *μ*M, Hep-3B: 100 *μ*M) or BIM (Sk-Hep-1: 780 nM, Hep-3B: 1170 nM). At 0, 24, and 48 h after scratching, cells were observed under phase-contrast microscope (Olympus, Tokyo, Japan). To measure the scratch widths and migration velocity, Image J software (version1.46) was used, and results were normalized vs the corresponding control groups.

### 2.7. Immunofluorescence Staining

To evaluate the quality of hepatocytic differentiation, immunofluorescence analysis was performed to visualize the expression of liver specific proteins in both HCC cell lines after treatment with 60 or 100 *μ*M CLA (Sk-Hep-1 and Hep-3B, respectively). Next, the samples were fixed in 4% paraformaldehyde, permeabilized (Triton™ X-100, Merck, Burlington MA,USA, 108634), blocked using BSA 1%, and incubated with primary antibodies against ALB (Abcam Cat NO: ab106582, 1 : 200) overnight at 4 °C in a moist chamber, followed by secondary antibody incubation for one h at 37 °C (Antigoat, Alexa Flour 488, Invitrogen, 1 : 1000). Then, the nuclei were counterstained with DAPI, washed with PBS, and visualized using a fluorescence microscope (Olympus, IX7).

### 2.8. ALB Secretion Assessment

The impact of CLA treatment on ALB secretion in Sk-Hep-1 and Hep-3B cells was measured by enzyme-linked immunosorbent assay (ELISA) (Bethyl Laboratories, Montgomery, TX, USA). Sk-Hep-1 and Hep-3B cells were treated with 60 or 100 *μ*M CLA in 6-well plates, respectively. The medium was refreshed every day. The supernatant was collected and ALB content measured by the ELISA kit according to the manufacturer's instructions.

### 2.9. Periodic Acid-Schiff (PAS) staining

The glycogen storage in Hep3B cells, after eight days of treatment with 100 *μ*M CLA, was visualized using periodic acid-Schiff (PAS) kit (Sigma-Aldrich Co. Missouri, USA) staining protocol. In brief, the cells were fixed in 4% paraformaldehyde for 20 min and then treated with periodic acid for 15 min. After washing with ddH_2_O, cells were incubated with Schiff's reagent for 5-20 min. The stored glycogen was visualized under a light microscope.

### 2.10. In Silico Data Analysis

The expression of HNF4*α* and its association with the EMT signatures [42] and differentiation markers were analyzed using TCGA-Biolink package under R software across 372 live carcinoma samples deposited in The Cancer Genome Atlas (TCGA) (https://portal.gdc.cancer.gov/). The protein-protein interaction was performed and analyzed using online version of STRING software (https://string-db.org/).

### 2.11. Data Analysis

All experiments were performed at least in three biological replicates. The data were analyzed using Prism software (version 6.07; GraphPad Software, San Diego, CA, United States). Since the distribution of the quantitative data was normal, comparison between the groups was evaluated using the ANOVA test and Fisher's least significant difference (LSD). The *p* < 0.05 was considered as statistically significant.

## 3. Results

### 3.1. CLA Treatment Reduces Cell Proliferation in Liver Cancer Cell Lines

The expression status of *HNF4a* in Hep-3B and Sk-Hep-1cell lines was initially evaluated by RT-qPCR, and data showed downregulation of *HNF4a* in both cell lines as compared to primary hepatocytes in 2D culture (Supplementary Figure 1(a)).

**Figure 1:**
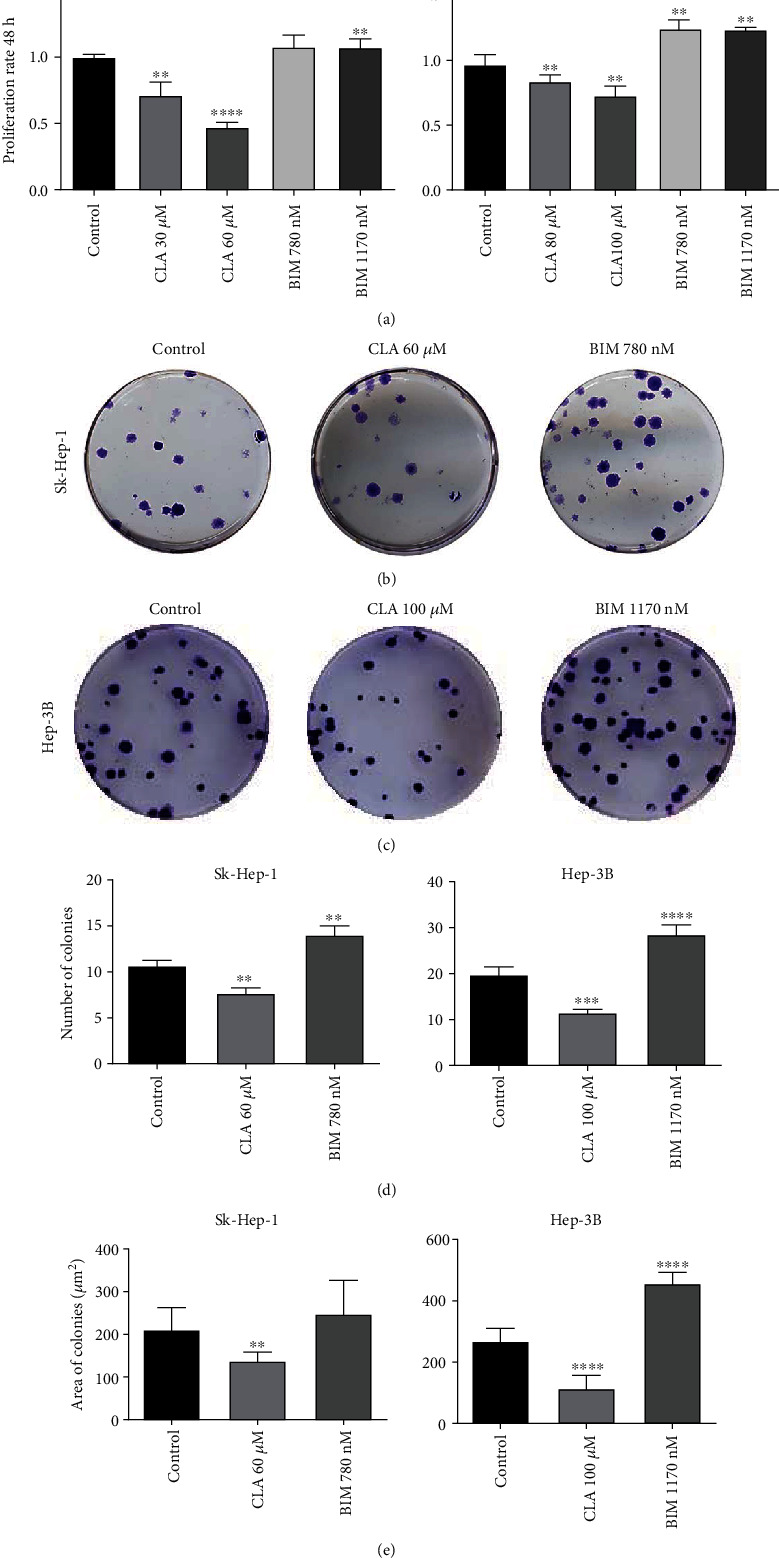
The proliferation rate and colony formation capacity of Hep-3B and Sk-Hep-1 cells after treatment with CLA or BIM for 48 h. (a) Sk-Hep-1 and Hep-3B cells which were treated with different concentrations of CLA (30, 60, 80, and 100 *μ*M) showed a significant reduction in proliferation rate in a dose-dependent manner as compared to the control groups (*n* = 3). Also the proliferation rates in both cell lines increased significantly after treatment with BIM. (b), (c), (d), (e) CLA treatment reduced the number of colonies and total area of them in both cell lines compared to the control groups. Also, the number and total area of colonies in BIM treated groups increased significantly compared to the control groups in both cell lines. Data are presented as the mean ± SD, *n* = 3 (^∗^*p* < 0.05, ^∗∗^*p* < 0.01, ^∗∗∗^*p* < 0.001, and ^∗∗∗∗^*p* < 0.0001).

Then, cell proliferation in Sk-Hep-1 and Hep-3B cells after treatment with CLA or BIM for 24 and 48 h was quantified. The cell proliferation rate for both cell lines significantly decreased in CLA treated groups as compared to the control groups in both time points in a dose-dependent manner by 30 and 55% for Sk-Hep-1 and 20 and 25% for Hep3B cells (Figure 1(a)), whereas BIM treatment significantly increased cell proliferation rate in both cell lines. Furthermore, Sk-Hep-1 and Hep-3B cells which were treated with 60 and 100 *μ*M CLA, respectively, showed a marked reduction in cell proliferation rate compared to the 30 and 80 *μ*M CLA after 48 h. This data indicated that CLA treatment reduced the proliferation rate in both HCC cell lines in a dose- and time-dependent manner and at both time points (Figure 1(a) and Supplementary Figure 1(b)).

### 3.2. CLA Treatment Attenuates Colony Formation Capacity in HCC Cell Lines

To evaluate colony formation capacity of both cell lines in terms of number and total surface area of emerged colonies, the plated cells were treated with an optimized concentration of CLA and BIM for each cell line (Figures 1(b) and 1(c)). Data showed that the number of colonies in CLA treated groups significantly decreased in both cell lines, while BIM treatment augmented colony formation capacity compared to the control groups in both cell lines (Figure 1(d)). Moreover, CLA treated colonies had a smaller surface area compared to the control and BIM treated groups in both cell lines (Figure 1(e)). BIM treatment in both cell lines resulted in more extended colonies in comparison with control groups, respectively. Altogether, CLA treatment considerably changed the colony-forming capacity of both HCC cell lines, and the number and surface area of the colonies were decreased remarkably.

### 3.3. CLA Treatment Induced Downregulation of EMT-Related Genes

**Figure 2:**
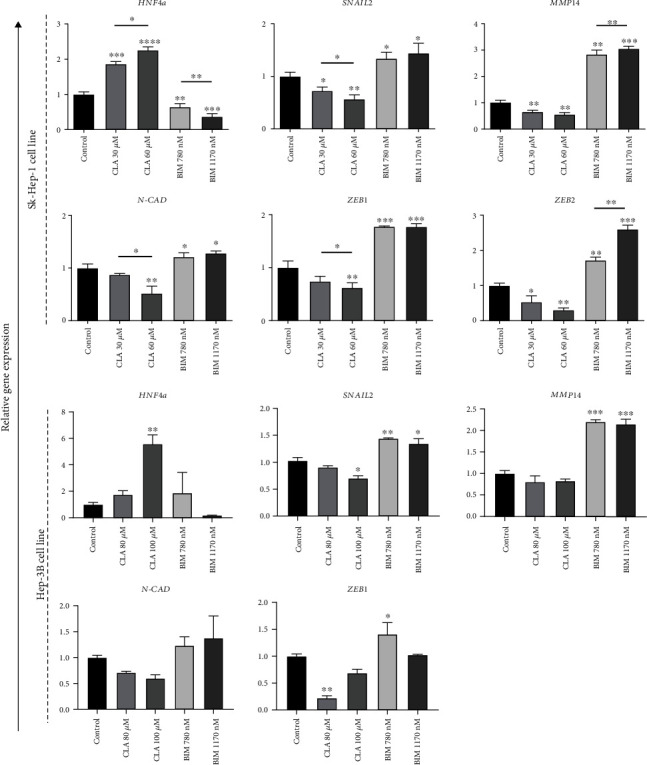
CLA treatment attenuated the expression of EMT related genes. The expression level of *HNF4a* increased after treatment with CLA in a dose-dependent manner, whereas the mRNA levels of EMT associated markers, e.g., *SNAIL2*, *ZEB1*, *MMP14*, and *N-CAD*, decreased in both Sk-Hep-1 and Hep-3B cell lines compared to the control groups. Downregulation of EMT-related genes in SK-Hep-1 cells was shown in a dose dependent manner. The expression pattern of the mentioned genes changed in opposite direction after treatment with BIM and upregulated in both cell lines. Also, the expression values showed dose dependent manner mostly in Sk-Hep-1 cells. Data are presented as the mean ± SD, *n* = 3 (^∗^*p* < 0.05, ^∗∗^*p* < 0.01, ^∗∗∗^*p* < 0.001, and ^∗∗∗∗^*p* < 0.0001).

The expression of *HNF4a*, a central regulator of hepatocytic differentiation, *SNAIL2*, *ZEB1*, *ZEB2*, and *CDH2 (N-CAD)*, EMT-related genes, and *MMP14*, a cell invasion marker, was assessed in both Sk-Hep-1 and Hep-3B cells after 48 h treatment with CLA and BIM at optimized concentration for each cell line. CLA treatment enhanced the expression of *HNF4a* in a dose-dependent manner in both cell lines as compared to the control groups. The upregulation of *HNF4a* was more than 2- to 6-fold in Sk-Hep-1 and Hep-3B cells, respectively, after treatment compared to the control groups. Regarding EMT-related genes, CLA treatment decreased the expression levels of *SNAIL2*, *ZEB1&2*, *MMP14*, and *N-CAD* in a dose-dependent manner, whereas BIM treatment induced the opposite in Sk-Hep-1 and Hep-3B cells (Figure 2). The expression of *ZEB2* was reduced in CLA treated SK-Hep-1 cells but was essentially undetectable in Hep3-B cells (Figure 2). Protein-protein interaction (PPI) between HNF4*a* and EMT-related proteins highlighted strong association between HNF4*a* and CDH1, SNAI1, SNAI2, and ZEB2. Correlation coefficient analysis between expression of the HNF4*a* and top 50 epithelial and mesenchymal genes across liver carcinoma samples was performed [42]. These results indicate a strong negative association (*R* = −0.4) between *HNF4a* upregulation and reduction of mesenchymal-related genes involved in EMT program. These results highlighted that induction of *HNF4a* might suppress mesenchymal phenotype and reduce metastatic capacity of liver carcinoma cells.

### 3.4. CLA Treatment Reduced the Migration Capacity of Both HCC Cell Lines

**Figure 3:**
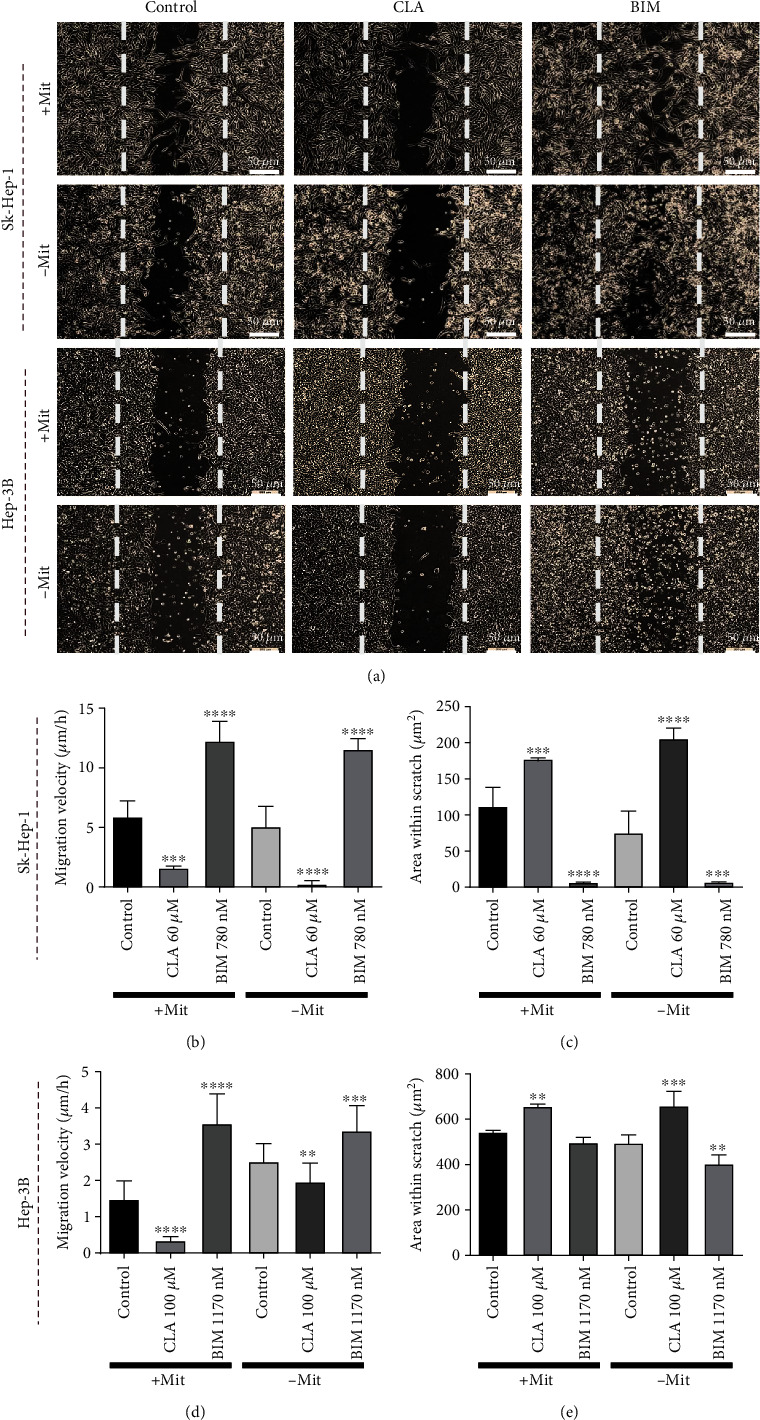
CLA treatment inhibited in vitro migration of HCC cell lines. (a), (b), (d) Scratch assay analysis showed that CLA treatment (Sk-Hep-1: 60 *μ*M, Hep-3B: 100 *μ*M) reduced the migration velocity of both HCC cell lines with or without mitomycin C treatment as compared to the control groups. However, BIM treated groups (Sk-Hep-1: 780 nM, Hep-3B: 1170 nM) showed enhanced motility in both experimental groups. (c), (e) The scratched areas that remained vacant were larger in CLA treated groups in comparison with control groups in both cell lines. BIM treatment in both cell lines resulted in an enhanced motility of cells and covering of almost all scratched areas. Data are presented as the mean ± SD, *n* = 3 (^∗^*p* < 0.05, ^∗∗^*p* < 0.01, ^∗∗∗^*p* < 0.001, and ^∗∗∗∗^*p* < 0.0001).

Sk-Hep-1 and Hep-3B cells migration capacity was assessed after treatment with CLA and BIM for 48 h. The migration velocity of both cell lines was significantly reduced in CLA treated groups compared to control groups (Figures 3(a), 3(b), and 3(d)). Moreover, comparison of the total scratch area revealed that in CLA treated cells, the vacant area in the dish after 48 h was larger compared to the control groups in both mitomycin treated (+Mit) and non-treated (-Mit) cells (Figures 3(c) and 3(e)). In contrast, BIM treatment notably enhanced the migration capacity of the both cell lines as compared to the control groups (Figure 3).

### 3.5. CLA Improved Hepatocytic Differentiation of Hep-3B Cells through HNF4*α* Activation

Since the Sk-Hep-1 cells are stromal hepatic cancer cells and originated from endothelial cells, hepatocytic differentiation analysis and functional evaluations were presented only on Hep-3B cells in the manuscript. To assess the effects of CLA on the induction of differentiated phenotype of Hep-3B cell line, expression levels of albumin in CLA treated cells were analyzed eight days post treatment.

**Figure 4:**
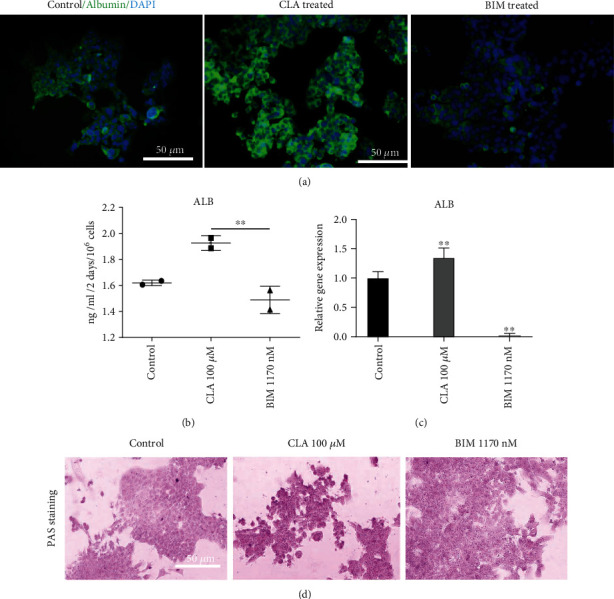
HNF4*α* induction resulted in improved hepatocytic differentiation of Hep-3B cells after CLA treatment. (a) Immunofluorescence staining revealed the remarkable expression of ALB in the CLA treated Hep-3B cells compared to the BIM treated and control group. (b) CLA treatment increased ALB secretion from Hep-3Bcells. (c) The relative mRNA expression of the *ALB* gene was increased after treatment with CLA in Hep-3B cells compared to the control group. (d) PAS staining showed glycogen accumulation in CLA treated cells as compared to BIM treated group. PAS: periodic acid-Schiff staining. Data are presented as the mean ± SD, *n* = 3 (^∗^*p* < 0.05 and ^∗∗^*p* < 0.01).

Significantly enhanced expression of ALB was shown in CLA treated Hep-3B cells, while BIM treatment decreased the expression level of this protein (Figure 4(a)). ELISA assay confirmed that CLA treated Hep-3B cells significantly secreted more ALB rather than BIM treated cells and respective control group (Figure 4(b)). In line with ALB protein expression level, *ALB* gene expression increased after treatment with 100 *μ*M CLA compared to the control group (Figure 4(c)).

To perform more assessments in terms of differentiation evaluation in CLA treated Hep-3B cells, glycogen storage was also evaluated. Glycogen storage was notably enhanced following CLA treatment as shown by using PAS staining method. On the contrary, BIM treatment decreased glycogen storage compared with the control group (Figure 4(d)). Supplementary Figure 2 presents the hepatocytic differentiation analysis for Sk-Hep-1 cells after treatment with CLA. This treatment improved ALB expression based on IF staining. Moreover, CLA treatment significantly upregulated *ALB* and *Cyp3A4* mRNA expression in Sk-Hep-1 cells.

### 3.6. In Silico Data Analysis Showed Strong Association between HNF4*a* and Specific Genes

**Figure 5:**
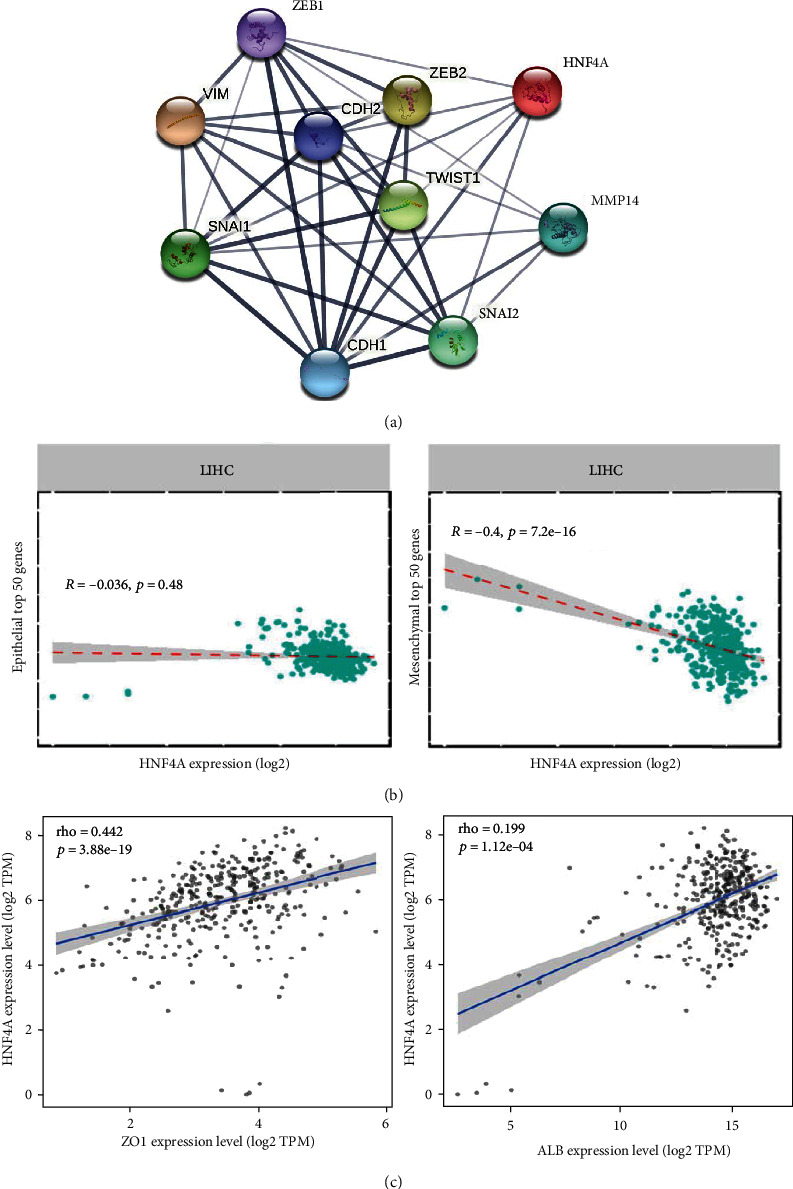
In silico data analysis. (a) Analysis of protein-protein interaction (PPI) between HNF4*a* and classical EMT-related proteins highlighting a strong association between HNF4*a* and CDH1, SNAI1, SNAI2, and ZEB2. (b) Correlation coefficient analysis between expression of HNF4*a* and top 50 epithelial and mesenchymal genes derived from (PMID: 25214461) across liver carcinoma samples (*n* = 372). These results indicate a strong negative association (*R* = −0.4) between the upregulation of *HNF4a* expression and the reduction of mesenchymal-related genes involved in EMT program. Compared to the mesenchymal genes, a weak negative association (*R* = −0.036) was observed between *HNF4a* expression and epithelial genes. These results highlighted that induction of *HNF4a* might suppress the mesenchymal phenotype and deteriorate the metastatic capacity of liver carcinoma cells. (c) The scatter plot depicts positive association between *HNF4a* and the expression levels of *ZO1* and *ALB* across 372 liver carcinoma samples from TCGA.

Protein-protein interaction (PPI) between HNF4*a* and classical EMT-related proteins highlighted a strong association between HNF4*a* and CDH1, SNAI1, SNAI2, and ZEB2 (Figure 5(a)). Correlation coefficient analysis between expression of HNF4*a* and top 50 epithelial and mesenchymal genes derived from (PMID: 25214461) across liver carcinoma samples (*n* = 372) indicated a strong negative association (*R* = −0.4) between the upregulation of *HNF4a* expression and the reduction of mesenchymal-related genes involved in EMT program (Figure 5(b)). Compared to the mesenchymal genes, a weak negative association (*R* = −0.036) was observed between *HNF4a* expression and epithelial genes. These results highlighted that induction of *HNF4a* might suppress the mesenchymal phenotype and deteriorate the metastatic capacity of liver carcinoma cells. The scatter plot depicts positive association between *HNF4a* and the expression levels of *ZO1* and *ALB* across 372 liver carcinoma samples from TCGA (Figure 5(c)).

## 4. Discussion

HNF4*α* is a liver-enriched TF that plays important roles including in gluconeogenesis and lipid metabolism [43, 44]. Numerous studies have shown that *HNF4α* expression is reduced in HCC patients in a stage dependent manner. Interestingly, the upregulation of *HNF4α* in cancer cells has been shown to be strongly associated with tumorigenesis suppression *via* induction of differentiation [45–47]. Overexpression of *HNF4α* is associated with a reduced proliferation rate and regulated expression of genes involved in the control of hepatocyte cell cycle [48]. Therefore, restoring the expression of *HNF4α* could be an influential milestone to reverse the HCC phenotype. Various molecular mechanisms control the expression of this TF *at* different levels, including epigenetic, transcriptional and post-transcriptional modifications [45, 49, 50]. Thus, several strategies have been employed to induce *HNF4a* overexpression in cancer cells using long-non coding RNAs, premade DNA vectors for *HNF4a*, miRNAs, small molecules, recombinant proteins, and growth factors [47, 51–60]. Recently, natural compounds have drawn much attention in the discovery and development of novel anticancer agents [61]. Natural compounds are bioactive ingredients produced by living organisms like animals, plants, fungi, and microorganisms that can selectively regulate signal transduction pathways and epigenetic mechanisms to modulate gene expression [62]. Studies have shown that CLA as a natural compound mainly found in ruminant products induces a decreased proliferation rate of cancer cells [63]. The multiple anticancerous effects of CLA were shown in a variety of cancers including HCC1. The literature was found that CLA exerts anticancerous features though different mechanisms including ER stress, autophagy, and PPAR *γ*. Our finding revealed that CLA could reduce cancerous phenotypes such as proliferation and colony formation, which were discussed in the following section [64–66].

Considering the association between the overexpression of *HNF4a* and reduction in cancerous phenotype of HCC cells, in the present study through a differentiation therapy approach, we investigated the effect of *HNF4a* induced expression in HCC cell lines after treatment with CLA, the natural ligand of *HNF4a*. Hep-3B as primary and Sk-Hep-1 as stromal liver cancer cell lines were assessed to show whether CLA treatment can reduce various cancerous features including proliferation rate, colony formation, and migration capacity. Our results showed that CLA treatment reduced proliferation rate, colony formation capacity, and migration of cancerous cells, whereas the expression of EMT-related genes was downregulated in a dose-dependent manner, while ALB production and glycogen storage capacity significantly increased. Results of a study demonstrated that the two isomers of CLA, trans10, cis12 (t10, c12), and c9,t11 have essential roles in growth inhibition in colon and prostate cancers. Treatment of Caco-2 cells with c9,t11 and t10,c12 isomers changed the expression pattern of lipid metabolism-related genes. Moreover, CLA treatment inhibited cell proliferation in breast cancer cells, and at the cytostatic concentration, CLA treatment caused cell cycle arrest in G1 [67]. Furthermore, in another study, the expression of *HNF4a* was upregulated after treatment with t10,c12 CLA [28]. However, the mechanism of CLA action on cell proliferation in various cancer cells was not clearly defined [68]. Correlation coefficient analysis between the expression of HNF4*a* and top 50 epithelial and mesenchymal genes across liver carcinoma samples demonstrated a strong negative association between the upregulation of *HNF4a* expression and reduction of mesenchymal-related genes involved in EMT process. Our data were also supported by in silico data and were in line with other studies [42].

The results suggested that CLA treatment may significantly reduce invasiveness capacity of cells through the reduction of EMT markers at the transcription level (Figure 2). On the other hand, substantial experimental evidence supports the contribution of hepatocytes that undergo EMT that form myofibroblasts in the injured liver. Therefore, it seems that CLA treatment can also prevent progression of liver fibrosis through EMT suppression [69].

The expression of *HNF4a* isoforms is tissue specific, and the liver expression pattern of *HNF4a* was remarkably changed during HCC progression. In this study, c9,t11 CLA isomer is used to induce *HNF4a* expression. Our results showed that CLA treatment of Sk-Hep-1 as the most invasive and endothelial tumor, and Hep-3B as the most undifferentiated HCC cell line, could reduce the proliferation rate as well as the number and size of colonies in a dose- and time-dependent manners. Some studies have shown a negative feedback loop between *HNF4a* and EMT-related genes [9, 70]. In our study, CLA treatment significantly increased the expression of *HNF4a* while inducing a significant down-regulation of EMT-related genes such as *Snail* in both cell lines. Furthermore, CLA treatment reduced the invasiveness of HCC cells and improved their hepatocytic differentiation phenotype, i.e., ALB secretion and glycogen storage.

The reduction of CLA in NAFLD and diabetic obese patients demonstrated the correlation between CLA and regulation of energy metabolism and maintenance of metabolic homeostasis in the liver [71]. CLA can also induce significant changes in the fatty acid profile of the liver [72]. In our study, we found that CLA treatment can also regulate the metabolic activity of cancerous cells and induce the expression of ALB and improve glycogen storage capacity. Altogether, our results, in correlation with our in silico findings, indicated the importance of *HNF4a* in mediating the EMT and MET in HCC cells as supported by the effects of CLA treatment on hepatocytic differentiation of HCC cells and the reduction of their cancerous features. Altogether, these results suggested that CLA might be used as a novel and natural differentiation inducing component for liver carcinoma cells. Our findings were acquired from *in vitro* experiments, and in near future we will evaluate such inhibitory effects of CLA on animal models to provide more reliable evidence to further clinical investigation.

## Data Availability

Data is available on request.

## Supplementary Materials

Supplementary Materials Supplementary Figure 1: (a) Relative mRNA expression of *HNF4α* in both HCC cell lines, Sk-Hep-1, and Hep-3B cells. The transcription level of *HNF4α* is very low in Hep-3B and Sk-Hep-1 cells. The expression of *HNF4α* was normalized to primary hepatocytes. (b) The proliferation rate of Sk-Hep-1 and Hep-3B cells was measured after treatment with different concentrations of CLA and BIM in two time points 24 h and 48 h. Treatment with CLA reduced the proliferation rate in a dose dependent manner in Sk-Hep-1 cells at both time points. Hep-3B cells also showed significant decrease in proliferation rate after treatment with CLA at both time points. BIM treatment induced proliferation rate in both cell lines at both time points. Data are presented as the mean ± SD, *n* = 3 (∗*p* < 0.05, ∗∗*p* < 0.01, ∗∗∗*p* < 0.001, and ∗∗∗∗*p* < 0.0001). Supplementary Figure 2: (a) Immunofluorescence staining induced the expression of ALB in CLA treated Sk-Hep-1cells as compared to the BIM treated and control groups. ALB expression is suppressed after treatment with BIM. (b) The relative mRNA expression of the *ALB*, *E-Cad*, and *CYP3A4* genes was increased after treatment with CLA in Sk-Hep-1cells as compared to the control group. BIM treatment resulted in significant decrease in the *E-Cad* and *ALB* genes. Data are presented as the mean ± SD, *n* = 3 (∗*p* < 0.05, ∗∗*p* < 0.01, ∗∗∗*p* < 0.001, and ∗∗∗∗*p* < 0.0001). Supplementary Table 1. The list of primers used in this study.
