# Supplementary material for: Conjugated Linoleic Acid Treatment Attenuates Cancerous features in Hepatocellular Carcinoma Cells
Source: Stem Cells Int. 2022 Sep 12;2022:1850305. doi: 10.1155/2022/1850305 (PMC9484933; doi:10.1155/2022/1850305)
Supplement: Supplementary Materials — Supplementary Figure 1: (a) Relative mRNA expression of HNF4α in both HCC cell lines, Sk-Hep-1, and Hep-3B cells. The transcription level of HNF4α is very low in Hep-3B and Sk-Hep-1 cells. The expression of HNF4α was normalized to primary hepatocytes. (b) The proliferation rate of Sk-Hep-1 and Hep-3B cells was measured after treatment with different concentrations of CLA and BIM in two time points 24 h and 48 h. Treatment with CLA reduced the proliferation rate in a dose dependent manner in Sk-Hep-1 cells at both time points. Hep-3B cells also showed significant decrease in proliferation rate after treatment with CLA at both time points. BIM treatment induced proliferation rate in both cell lines at both time points. Data are presented as the mean ± SD, n = 3 (∗p < 0.05, ∗∗p < 0.01, ∗∗∗p < 0.001, and ∗∗∗∗p < 0.0001). Supplementary Figure 2: (a) Immunofluorescence staining induced the expression of ALB in CLA treated Sk-Hep-1cells as compared to the BIM treated and control groups. ALB expression is suppressed after treatment with BIM. (b) The relative mRNA expression of the ALB, E-Cad, and CYP3A4 genes was increased after treatment with CLA in Sk-Hep-1cells as compared to the control group. BIM treatment resulted in significant decrease in the E-Cad and ALB genes. Data are presented as the mean ± SD, n = 3 (∗p < 0.05, ∗∗p < 0.01, ∗∗∗p < 0.001, and ∗∗∗∗p < 0.0001). Supplementary Table 1. The list of primers used in this study. [file 1850305.f1.zip › Lavasani et al-R2-Supplementary Figure 1.pptx]

## Slide 1
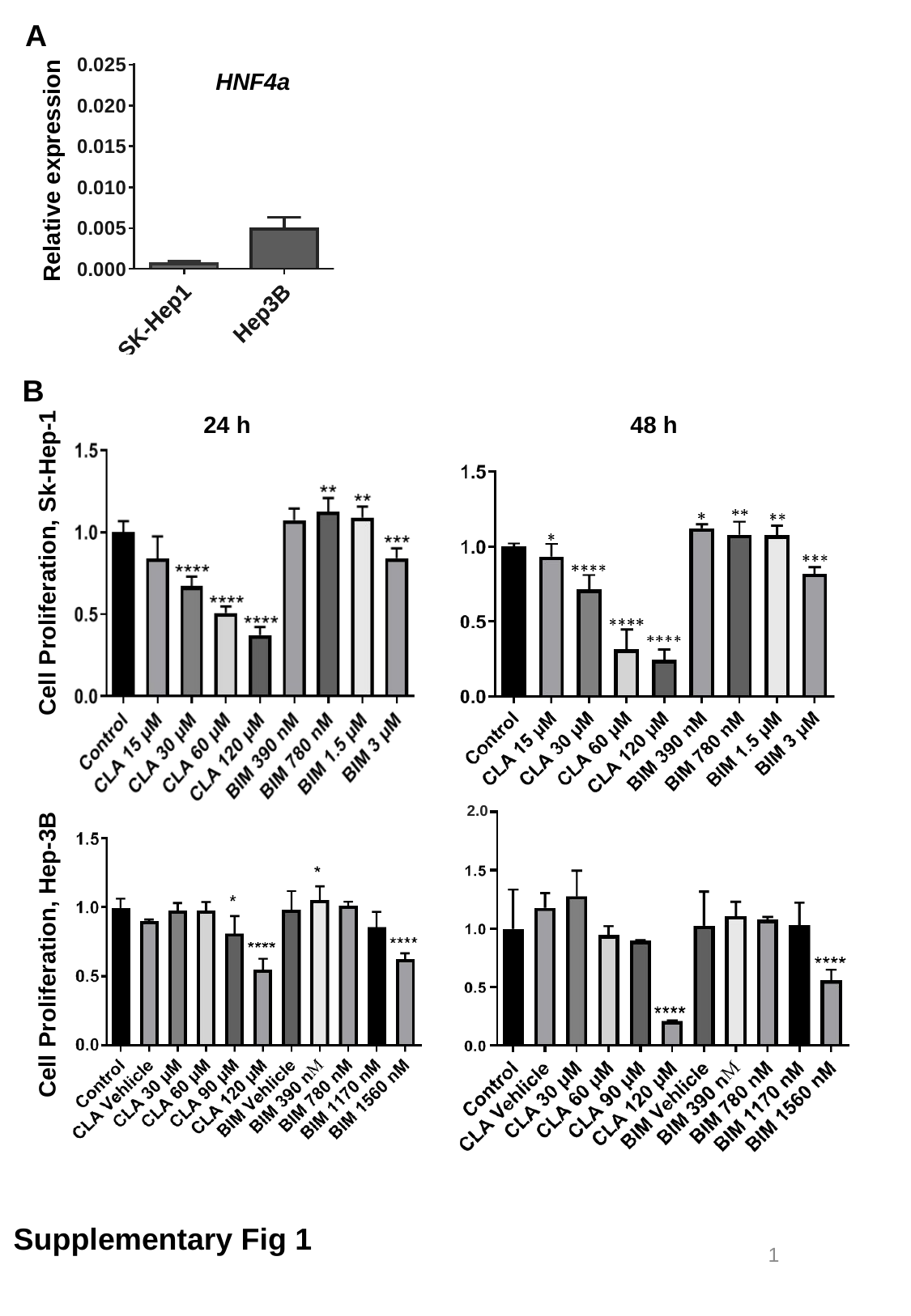

A
HNF4a
Relative expression
B
24 h
48 h
Cell Proliferation, Sk-Hep-1
2.0
Cell Proliferation, Hep-3B
Supplementary Fig 1
1
